# Rapid Nucleic Acid Extraction and Purification Using a Miniature Ultrasonic Technique

**DOI:** 10.3390/mi8070228

**Published:** 2017-07-21

**Authors:** Darren W. Branch, Erika C. Vreeland, Jamie L. McClain, Jaclyn K. Murton, Conrad D. James, Komandoor E. Achyuthan

**Affiliations:** 1 Nano and Micro Sensors Department, Sandia National Laboratories, Albuquerque, NM 87185, USA; kachyut@sandia.gov; 2 Imagion Biosystems, Inc., Albuquerque, NM 87106, USA; erika.vreeland@imagionbio.com; 3 MEMS Technologies Department, Sandia National Laboratories, Albuquerque, NM 87185, USA; jlmccla@sandia.gov; 4 Bioenergy and Defense Technologies Department, Sandia National Laboratories, Albuquerque, NM 87185, USA; jkmurto@sandia.gov; 5 Physics Based Microsystems Department, Sandia National Laboratories, Albuquerque, NM 87185, USA; cdjame@sandia.gov

**Keywords:** acoustic lysis, microfluidic, nucleic acids, extraction, purification, separation, Point-of-Care

## Abstract

Miniature ultrasonic lysis for biological sample preparation is a promising technique for efficient and rapid extraction of nucleic acids and proteins from a wide variety of biological sources. Acoustic methods achieve rapid, unbiased, and efficacious disruption of cellular membranes while avoiding the use of harsh chemicals and enzymes, which interfere with detection assays. In this work, a miniature acoustic nucleic acid extraction system is presented. Using a miniature bulk acoustic wave (BAW) transducer array based on 36° Y-cut lithium niobate, acoustic waves were coupled into disposable laminate-based microfluidic cartridges. To verify the lysing effectiveness, the amount of liberated ATP and the cell viability were measured and compared to untreated samples. The relationship between input power, energy dose, flow-rate, and lysing efficiency were determined. DNA was purified on-chip using three approaches implemented in the cartridges: a silica-based sol-gel silica-bead filled microchannel, nucleic acid binding magnetic beads, and Nafion-coated electrodes. Using *E. coli,* the lysing dose defined as ATP released per joule was 2.2× greater, releasing 6.1× more ATP for the miniature BAW array compared to a bench-top acoustic lysis system. An electric field-based nucleic acid purification approach using Nafion films yielded an extraction efficiency of 69.2% in 10 min for 50 µL samples.

## 1. Introduction

Extracting nucleic acid for identification of biological agents and diseases is a still a challenging requirement in Point-of-Care (POC) medical diagnostics. In particular, POC genomic testing would greatly benefit from technology capable of operating in minimalized settings with the same performance as a central lab. Next generation bioanalysis platforms require fast DNA extraction and purification methods that allow unbiased isolation of specific targets from a variety of microorganisms and biological sources. However, the process is complicated by the inherent complexities of biological samples, where background materials must be removed or minimized to manage interference and fouling in the microsystem. Two essential requirements of sample preparation methods are achieving efficient extraction and purification of nucleic acids without harsh chemicals while maintaining low system losses. These will enable rapid detection in a small format and move toward the same performance as a central lab.

Even current nucleic acid lysis and extraction techniques [1] require significant manual intervention and consumables leading to limitations that are especially relevant for the unattended, timely detection of biological agents or other microorganisms. There is a still continued reliance on large laboratory equipment such as centrifuges, gel electrophoresis units, and ultracentrifuges. Most laboratory extraction methods require chemical or enzymatic techniques that are labile, or need special handling such as temperature control, storage, and disposal. These factors further impede progress toward miniaturized autonomous detection. 

Recently, major advancements have been made using microfluidic techniques to enable fully integrated nucleic acid extraction systems [2]. Cellular disruption (i.e., lysis) methods can be classified as physical, chemical, thermal, enzymatic, and ultrasonic where each has its strength depending on the biological agent and quantity to be processed [3,4]. In particular, a chemical free technique has been developed to perform DNA extraction, purification using aluminum oxide membranes (AOM), and PCR analysis of *S. aureus* and *S. mutans* from saliva [5]. Chemical-based lysis methods are typically easier to integrate into microfluidic systems and are often combined with solid-phase DNA extraction and purification methods such as silica or sol-gels where washing is used for sample cleanup and elution [6,7]. Chemical-based lysis often involves the use of proteases or denaturants such as proteinase K to hydrolyze peptide bonds, which must be removed prior to PCR analysis through washing. A complete blood lysis and extraction approach based on proteinase K and paramagnetic beads has been demonstrated [8]. As an alternative to PCR, methods such as Loop-mediated isothermal amplification (LAMP) can achieve amplification and detection of DNA faster and more robustly than PCR. This technique has recently been used for blood analysis to provide viral and bacterial testing using chemical lysis and LAMP for real-time detection [9]. 

Thermal methods have also proven useful for releasing nucleic acids without introducing PCR inhibitors [10,11]. However, proteins will suffer denaturation due to disruption of hydrogen bonds and hydrophobic interactions at temperatures around 47 °C [12]. In contrast, ultrasonic waves provide excellent control over the delivery of acoustic energy into fluidic samples and thus span a wide range of effects such as particle manipulation [13,14,15], mixing [16], removing non-specifically bound proteins [17,18], sorting [15], and cellular lysis [3,19,20,21]. Historically, the ultrasonic lysing mechanism is proposed to occur from gaseous cavitation in which air bubbles rapidly form and collapse or cellular lysis occurs from shearing in the absence of cavitation or bubble formation at much lower power [22]. It is known that the cavitation threshold increases rapidly with frequency and is estimated to be in excess of 1000 W/cm^2^ at 1 MHz [23]. This suggests the lysing mechanism for high frequency transducers is through other mechanisms such as acoustic radiation pressure, shear forces, and to a lesser extent, localized heating. Recently, a low-intensity (0.1–1 W/cm^2^) acoustic focusing method has achieved selective lysis of cancer cells based on their biomechanical properties as related to their acoustic energy threshold [24,25].

Acoustic waves are an ideal lysing mechanism since they can deliver acoustic energy into sealed systems such as microchannels. They also avoid the use of harsh chemicals that interfere, inhibit, or introduce bias when using detection methods such as PCR unless the sample is purified. Purification adds additional steps that increase the time before a sample can be analyzed. High power acoustic transducers have proven powerful for disrupting cell membranes and spores for subsequent DNA analysis [3,20]. Though successful, these transducers required processing volumes that were not suitable for microfluidic systems. 

Efforts to miniaturize an acoustic lysis system for microfluidic applications have led to the use of thin-film piezoelectric-based transducers. Applications include thin films of zinc oxide (ZnO, *k_t_*^2^ = 5%) [21,26] or piezoceramics such lead zirconate titanate (PZT, *k_t_*^2^ = 50%) [24,27]. It is difficult to fabricate ZnO transducers below 300 MHz due to the upper limit of attainable film thickness and thin-film reproducibility issues. Transducers based on PZT generate acoustic waves more efficiently due to their large electromechanical coupling coefficient (*k_t_*^2^) but are limited to the low-frequency domain (*f* < 25 MHz, λ/2 <0.1 mm, *v* = 5000 m/s). Most of the applied power is converted into heat rather than mechanical motion and ultimately the temperature of the fluid rises in the microchannel. In some cases, the lysing process may benefit from heat generation though it is difficult to determine the role of each mechanism without thermal control during lysis. Applications of surface acoustic wave (SAW) devices have been investigated previously for mechanical manipulation of samples using the phenomenon known as acoustic streaming [22,28,29]. Notably, surface acoustic waves (SAWs) enable a wide range of sample preparation activities: removal of non-specifically bound proteins on microarrays [17], lysis via high-speed cellular collision [30], lysis for PCR analysis using acoustic field manipulation [31], and combined with microfluidic antibody capture (MAC) techniques for protein analysis [32].

In our previous work, a low-power lysis approach was developed with negligible temperature rise in the microchannels for rapid extraction of *Mycobacterium tuberculosis* (MTB) DNA [33]. In this study, we investigated three types of DNA extraction methods integrated with a miniature bulk acoustic wave (BAW) transducer array on a disposable laminate device. The BAW transducer array was fabricated using 36° Y-cut (90°, 90°, 36°) lithium niobate which generated and coupled acoustic waves into disposable microfluidic cartridges fabricated using plastic laminates. The electromechanical coupling (*k_t_*^2^) for 36° Y-cut lithium niobate is 46%, where the transducer operated from 50–70 MHz (see Appendix A). A transmission line model and the finite-element method (FEM) were used to design the transducer and maximize acoustic coupling into disposable microchannels [33]. DNA was extracted and purified using three approaches: (1) silica-based sol-gel filled microchannels; (2) nucleic binding paramagnetic beads; and (3) Nafion-coated gold electrodes, where the extraction efficiency was compared for each method.

## 2. Materials and Methods

### 2.1. Transducer Fabrication

**Figure 1:**
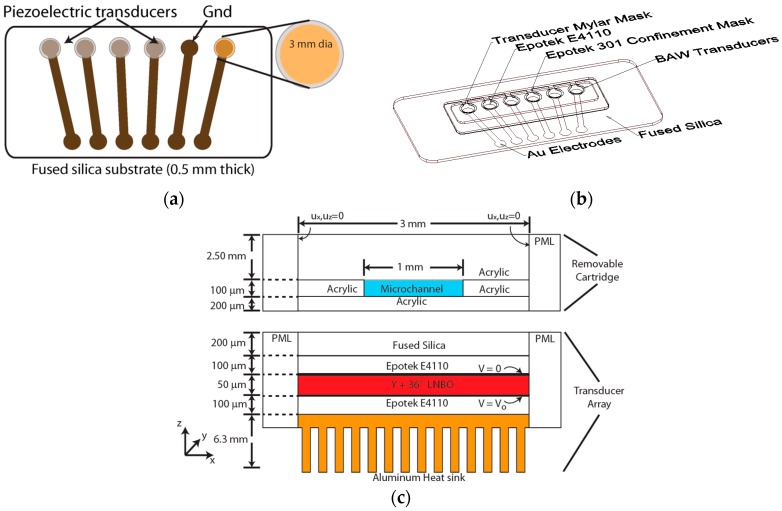
Fabrication of the miniature bulk acoustic wave (BAW) transducer array. (**a**) 36° Y-cut lithium niobate substrates were bonded to the fused-silica substrate using conductive epoxy; (**b**) Mylar^®^ masks were applied prior to encapsulation to control the placement of the conductive epoxy; and (**c**) The cross-section of the transducer and disposable cartridge assembly.

The transducers were fabricated using 36° Y-cut lithium niobate (Boston Piezo-Optics, Bellingham, MA, USA), approximately 50 µm thick and 3 mm in diameter [33]. Gold (Au) electrodes were 0.2 µm thick with a 0.2 mm hold back from the edge of the substrates. Electrical connections to the piezoelectric substrates were made by depositing gold electrodes and contact pads onto fused silica substrates (20.5 mm × 44.8 mm × 0.5 mm) obtained from University Wafer (South Boston, MA, USA) (Figure 1a). A metal shadow mask was used to pattern a 0.2 µm thick Au electrode pattern onto the fused silica. The piezoelectric substrates were bonded to gold contact pads patterned on the fused silica substrate using a 100 µm thick layer of Epotek 4110 conductive epoxy (Epotek Technology, Billerica, MA, USA). The placement of the conductive epoxy was controlled by using a 4 mil Mylar^®^ masking layer cut using a VersaLaser VLS3.50 system (Universal Laser Systems, Scottsdale, AZ, USA) (Figure 1b). The E4110 epoxy was cured at 150 °C for 15 min. The transducers were bonded and checked for short-circuits prior to encapsulation. A confinement Mylar^®^ mask was attached directly to the transducer Mylar^®^ mask. The backside of each transducer was masked during the encapsulation process using PDMS cylinders (2.5 mm dia., 3 mm height). The encapsulating epoxy was prepared using 5:20 (*w/w*) mixture of Epotek 301A and Epotek 301B and cured at 60 °C for 1 h. After curing, the PDMS cylinders were removed and the region was filled with Epotek 4110 conductive epoxy. An Al heatsink was bonded above the transducers using a 10:1 (*w/w*) mixture of Epotek 4110A and Epotek 4110B (Epotek Technology, Billerica, MA, USA) conductive epoxy and cured for 15 min at 150 °C. A cross-section of the transducer and cartridge assembly is shown in Figure 1c.

### 2.2. Cell Sample Preparation and Cellular Lysis Quantitation

*Escherichia coli* (*E. coli*) cultures (ATCC 10798) were purchased and cultivated in BBL™ Mueller Hinton II Broth, Cation Adjusted (Becton Dickinson and Company, Sparks, MD, USA), following standard methods. *E. coli* was incubated at 37 °C and shaken in a water bath at 120 RPM, and growth was tracked by monitoring the change in optical density of the suspension at 600 nm (OD_600_) using a DU-800 spectrophotometer (Beckman Instruments, Inc., Fullerton, CA, USA). Cells were grown to log phase, harvested, washed twice in 1× PBS (137 mM NaCl, 2.7 mM KCl, 4.3 mM Na_2_HPO_4_·7H_2_O, 1.5 mM KH_2_PO_4_) and re-suspended in 1× PBS at an average cell density of 5 × 10^8^ CFU/mL. The stock suspension was kept refrigerated and all experiments were completed within two hours of the initial sample preparation. A volume of 50 µL of cell suspension was pumped into the microfluidic laminate cartridges using a syringe pump (KD Scientific, Holliston, MA, USA). The lysing efficacy was measured by comparing viable cell counts before and after treatment using a plate counting method. Untreated and acoustically treated cells were grown on agar plates after flowing them through the microchannel lysis system and not by growing *E. coli* inside the microchannels themselves. A serial 10-fold dilution of the sample was performed in sterile 1× PBS within ten minutes of the lysis experiments. Aliquots of 20 µL were spotted in triplicate onto Difco™ plate count agar (Becton Dickinson and Company, Sparks, MD, USA) and incubated at room temperature for 24 h. The percent of viable cells was calculated using 100 × C_treated_/C_0_, where C_0_ is the average viable cell count prior to sonication and C_treated_ is the average viable cell count after sonication. 

Lysing efficacy was quantified by measuring the ATP liberated from acoustically treated samples and comparing it to the measured ATP of untreated samples. A firefly luciferase-based ATP Determination Kit was purchased from Molecular Probes (Eugene, OR, USA), and performed in a 96-well microplate format. The limit of detection of this kit is 0.1 pmol of ATP. Luminescence measurements were taken using a Berthold-Mithras LB 940 microplate reader (Berthold Technologies, Oak Ridge, TN, USA). ATP concentrations were calculated by fitting experimental data to a standard curve generated with known concentrations of ATP. Linearity of light output vs. ATP concentration was confirmed using ATP standard solutions over the range of 10^−10^–10^−6^ M. 

Positive lysis controls were performed by using a 20 kHz Sonicator 3000 bench top sonication system (Misonix, Farmingdale, NY, USA) using an acoustic finger and acoustic cup configurations. For the acoustic finger experiments, the tip was inserted into a 2 mL microcentrifuge tube containing a 1 mL sample using 12 W of applied power at 8% duty cycle as recommended by the manufacturer (pulse on time was 5 s, pulse off time was 60 s, 5 treatments, and total process time was 325 s). During the experiment, the sample container was submerged in an ice bath to dissipate heat generated by the acoustic transducer. In the acoustic cup configuration, 100 µL of sample in a closed 2 mL microcentrifuge tube was submerged into a water-filled cup, the base of which encapsulated a 45.7 mm diameter flat acoustic transducer. During the experiment, 58.5 W of power was applied at a 100% duty cycle (total process time = 21.5 min). Heat generated by the transducer was dissipated by circulating the water through a chiller for the duration of the experiment set at 23 °C.

**Figure A2:**
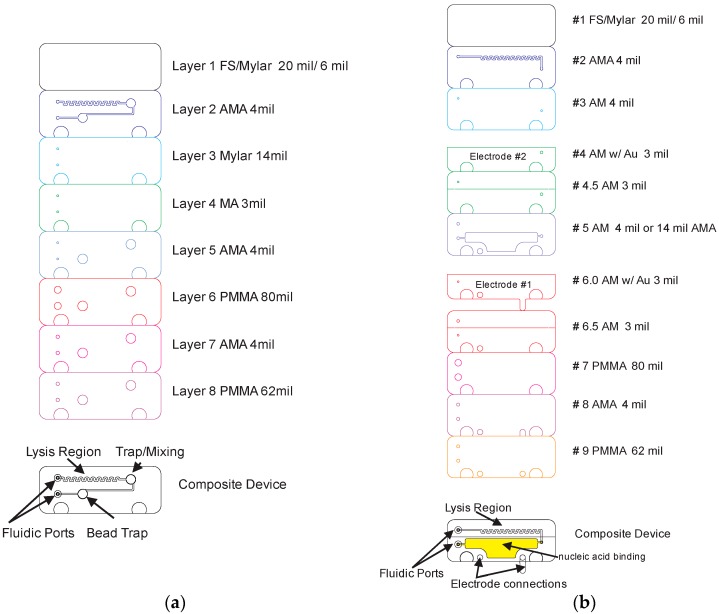
Acoustic lysis and nucleic acid extraction microchannel laminant layers, (**a**) Lysing, mixing, and DNA extraction cartridge using paramagnetic beads. FS: Fused silica, AMA: adhesive/Mylar^®^/adhesive, MA: Mylar^®^/adhesive, PMMA; (**b**) Nafion-coated gold (Au) electrodes captured nucleic acids by application of a DC bias. AM: Adhesive/Mylar^®^. Cells were introduced into to the lysis region followed by DNA capture, washing, and elution. The laminant thicknesses were specified in mils by the manufacturer, where 1 mil is 25.4 µm.

For the miniature BAW lysis array, the samples were: (1) flowed into the serpentine region and then stopped (static) and the transducers were driven, or (2) samples were flowed continuously (flow) across the serpentine region during lysis (Appendix B, Figure A2). An 8648A synthesized signal generator (Agilent Technologies, Santa Clara, CA, USA) and an RF power amplifier (Bell Electronics NW, Inc., Kent, WA, USA) were used to drive the piezoelectric transducer array. The input power was varied from 0 to 200 mW at 100% duty cycle and the sample flow rates ranged from 10 to 25 μL/min in order to maximize lysing efficiency while avoiding excessive heat generation and acoustic cavitation. Using a flow rate of 10 μL/min and the dimensions of the microchannel (width: 100 μm, height: 500 μm, length: 18.4 mm), the flow velocity was 3.3 mm/s and the dwell time (i.e., volume/flow rate) in the microchannel was 5.5 s.

### 2.3. Nucleic Acid Extraction Approaches with Acoustic Lysis

#### Sol-Gel Silica Bead Nucleic Acid Extraction

**Figure 2:**
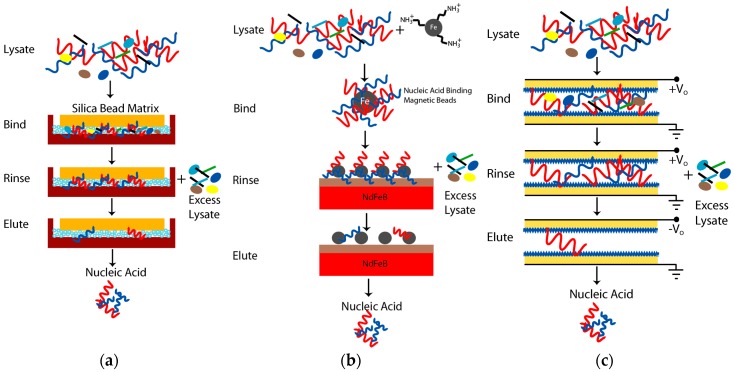
Nucleic acid extraction approaches. (**a**) Sol-gel silica bead matrix-based extraction; (**b**) Paramagnetic bead (ChargeSwitch^®^)-based nucleic acid extraction; (**c**) Nafion-coated Au electrodes for nucleic acid extraction.

It has been reported that using silica beads alone in microchannels was problematic due to compression of the particles during use whereas sol-gel filled microchannels lack mechanical stability [6]. Instead, the two approaches were combined to produce a stable matrix for DNA extraction. An injection method was used to fill microchannels with a sol-gel-bead matrix (Figure 2a). The microchannels were fabricated using wet etching of borosilicate and were 150 µm wide, 150 µm deep, and 332 mm in length (Micronit, PV Enschede, The Netherlands). This design was more compatible with processing higher sample volumes due to the deeper channel and increased surface area. The extraction volume was 7.5 µL. The sol was prepared by hydrolyzing a 27% *v/v* solution of tetraethoxysilane (TEOS) (Sigma-Aldrich, St. Louis, MO, USA) in water by the addition of 0.1% *v/v* HNO_3_ and heating to 60 °C for 10 min then 80 °C for 60 min with stirring at 200 rpm [6]. Silica beads with a diameter of 5 µm were added prior to gelation at a concentration of 200 mg of beads in 1 mL of sol. Gelation of the sol was carried out by increasing the temperature to 100 °C to achieve condensation under acidic conditions. Using 5 µm diameter silica beads minimized the settling problem when preparing the suspensions as compared to suspensions with larger silica bead diameters. The resulting sol-gel silica bead matrices bind DNA/RNA through an electrostatic interaction. The sol-gel silica bead matrix was hydrophilic since it was formed by the acid catalyzed condensation of the sol. The silica beads provided a large surface area for selective binding of DNA, while the sol-gel serves as a silica-based glue to hold beads in place during device operation. An injection method was used to introduce the sol-gel-bead matrix into the channel under pressure. The chamber outlet was plugged ~90% to allow some fluid to exit, while confining most of the beads in the channel. After filling, the chamber was dried at 120 °C for 24 h.

Samples were injected into sol-gel filled microchannels using a syringe pump (PHD 2000, Harvard Apparatus, Holliston, MA, USA) with a 100 µL Hamilton gas tight syringe (Hamilton, Las Vegas, NV, USA). The syringe pump was connected to the sol-gel silica bead filled microchannels using 1/16” OD and 20 mil ID PEEK tubing (IDEX, Oak Harbor, WA, USA). NanoPort Assembly (IDEX, Oak Harbor, WA, USA) connectors were bonded to the microchannel inlet and outlet ports for the tubing connections.

The extraction process was initiated by rinsing the microchannels with MeOH to activate the silica. The microchannels were flushed with TE (10 mM Tris, 1 mM EDTA, titrated to pH 7.6 with HCl) and 6 M guanidine hydrochloride between extraction experiments. All the solutions were prepared in 18 MΩ·cm water. Loading was performed using 25 µL of DNA (250 fmol) in 6 M guanidine hydrochloride, which was flowed through the microchannels at 2 µL/min. The wash step was performed using 25 µL of 80% 2-propanol. This removed any excess guanidine hydrochloride and contaminants from the microchannels. DNA was eluted from the solid phase by flowing TE buffer through the microchannels. The load, wash, and eluent were collected in microcentrifuge tubes and analyzed using DNAQF DNA quantitation fluorescent assay kit (Sigma-Aldrich, St. Louis, MO, USA). The fabrication process for the acoustic lysis combined with paramagnetic beads (ChargeSwitch^®^) and Nafion-based nucleic acid extraction cartridges are detailed in Appendix B.

## 3. Results and Discussion

### 3.1. Miniature BAW Transducer Array and Cellular Lysis

**Figure 3:**
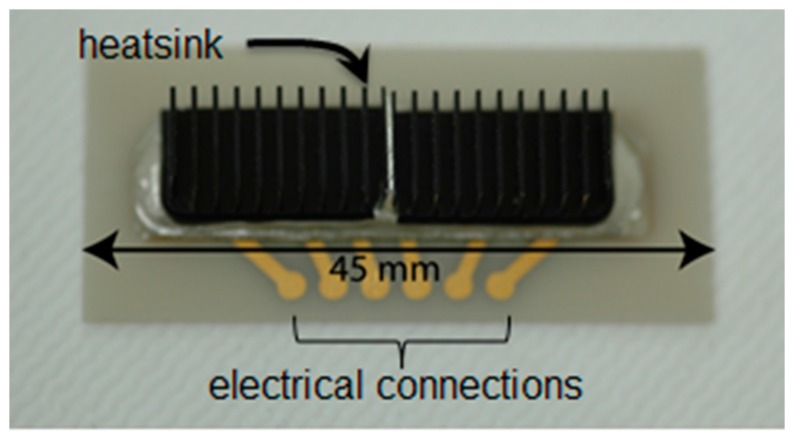
The final assembled four-channel BAW array. The opposite face is a flat plate of aluminum nitride (AlN).

The details of the transducer operation and performance were cited previously [33]. Briefly, 36° Y-cut lithium niobate (90°, 90°, 36°) (*z, x, z*) excites a quasi-longitudinal wave with an electromechanical coupling of *k_t_* = 0.46 with an unloaded acoustic velocity of 7340 m/s. Excitation of the two shear modes was negligible in this propagation direction. In Figure 1c, the BAW transducer array was placed in physical contact with the disposable microfluidic cartridges, which are shown in Appendix B, Figure A2. The completed BAW array is shown in Figure 3. This design produced minimal heating of the fluid in the microchannel, making it suitable for biological applications. The temperature of the fluid in the microchannels was monitored during the lysis experiments and did not exceed 6 °C above the ambient room temperature of 23 °C.

**Figure 4:**
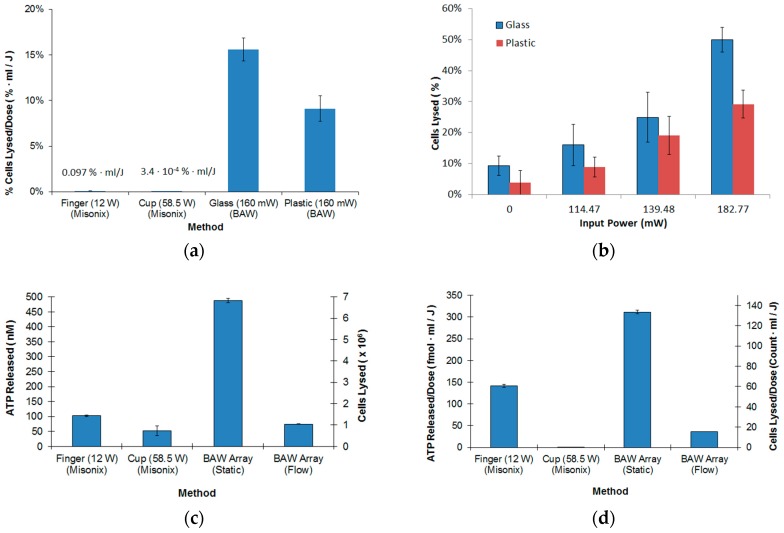
Lysing efficacy assessed by viable cell counting, ATP release, and energy dose per mL of sample volume. (**a**) Percentage of cells lysed per joule versus method; (**b**) BAW array cell lysis versus input power using plastic and glass bottom interfaces to the microchannels at 10 µL/min; (**c**) ATP released (nM) and cells lysed vs. method. For the BAW array, *static* refers to fill then treated for 30 s, and *flow* refers to the sample flowing through the microchannel at 10 µL/min. The ATP concentration for the untreated samples were 100 ± 12 pM; (**d**) ATP released/dose and cells lysed/dose vs. method. The dose was computed as the applied energy per 1 mL of sample volume. *n* = 5, reported as mean ± std. dev.

In Figure 4a, a comparison with a commercial lysis system is shown normalized to applied energy dose in joules. The finger method refers to the use of an acoustic probe inserted into the biological samples. This method was open to the environment and required an ice bath with 60 s rest periods to prevent sample degradation. The process used a pulse on time of 5 s, a pulse off time of 60 s, five treatments, and total process time of 325 s. The cup method refers to an acoustic bath that treats the samples inside conical tubes. Although the cup method was closed to the environment, a microfluidic flow option is not easily included. Static refers to fill then lyse and flow means the sample was pumped through the microchannels. In terms of cells lysed, the finger option was far more efficient than the cup, followed by the BAW array coupled to a glass, then plastic bottom laminate-based microchannels (Figure 4a). The percentage of *E. coli* lysed was nearly identical to the commercial system but it used 75× less power for the glass bottom microchannel. As expected, a laminate channel with a plastic bottom in contact with the BAW array did not couple acoustic pressure as efficiently into the microchannel. In this case, the performance was nearly identical to the cup method using 365× less power. Of significance is the dramatic reduction in treatment time, where only 20 s was required to achieve 50% lysis of the sample. The input power was computed as the amount of power the transducer received, compensating for losses and source mismatch. In terms of applied energy (power × treatment time), the BAW array used 225× less and 23,000× less than the finger and cup methods, respectively. 

In Figure 4b, the percentage of cells lysed was 5% to 20% higher for the microfluidic cartridges with glass bottoms coupled to the transducer compared to cartridges with plastic interfaces as input power was increased. This result was anticipated since glass has very low acoustic loss compared to plastic. However, this result demonstrates the ability to lyse cells using a completely plastic-based microchannel, which substantially simplifies fabrication complexity and cost.

The cup method had a large variation in the percentage of cells lysed that was a result of acoustic waves not being directed into the samples. In Figure 4c,d, the BAW array under the static (i.e., no flow) conditions released 6.1× more ATP than the finger method and 2.2× more ATP per joule. The cup option suffered from poor efficiency since it radiated acoustic waves into the water surrounding the samples rather than directly into the sample volume.

Under flow conditions, the BAW array performed similarly to the finger in terms of ATP release and cells lysed (Figure 4c). Improved cell lysis benefited from increased exposure time to the acoustic BAW array using a serpentine channel rather than a standard linear microchannel (Appendix B, Figure A2a). The flow rate was kept above 10 µL/min to minimize temperature rise in the microchannel. The additional path length provided an additional tuning parameter between lysing efficacy and the subsequent nucleic acid extraction. For both interface types, Figure 4b showed that increasing the applied power improved lysing efficiency, which required a balance between input power and heat generation by the transducer according to a thermal analysis [33].

The efficacy of the BAW microfluidic lysing system is very competitive with the commercial system, requiring much lower power input (i.e., ~200 mW as compared to the commercial system operating at 12 W to 58.5 W). The performance gain of the microfluidic lysing system was attributed to the operating frequency and hence the number of sonication cycles the cells experience during treatment. The number of sonication cycles per cell for the 54 MHz BAW array was estimated using the dwell time (i.e., volume/flow rate) of the fluid in the microchannel, which was 5.5 s. This gives an estimated sonication cycle per cell of 5.5 s × 54 × 10^6^ cycles/s = 3 × 10^8^ cycles per cell. In comparison, the sonication cycles for the commercial system was 25 s × 20 × 10^3^ cycles/s = 5 × 10^5^ cycles per cell. This suggests that more sonication cycles improved the cellular lysis. However, adding more sonication cycles using a low frequency system (i.e., 20 kHz Sonicator 3000) can cause problems such as an increased temperature and cavitation. Other contributing factors to performance gain include: properties of the cells, scattering, acoustic absorption, thermal effects, particle forces and concentration, and media compressibility [34]. The error bars at each data point indicate variation in viable cell counts; however, this was independent of the lysis method and rather a result of the assay method (Figure 4b). It was attributed to the number of serial dilutions that were required to obtain appropriate plate counts, resulting in a 1:100,000 dilution, which amplified small variations in counting.

### 3.2. Sol-Gel Packed Microchannels

**Figure 5:**
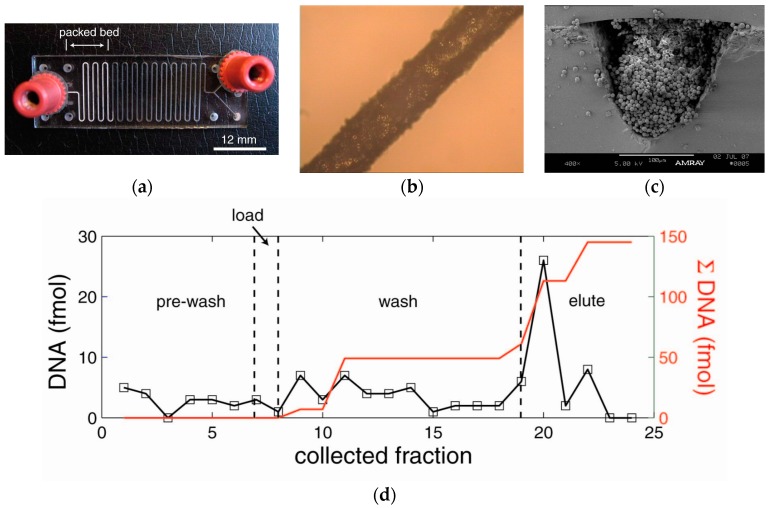
DNA extraction device and performance (**a**) Sol-gel packed microchannel; (**b**) Dried sol-gel-bead matrix cured and packed in the microchannel; (**c**) Cross-section of the packed bed section of sol-gel immobilized silica beads (5 µm) in channel; (**d**) collected fractions over time for the DNA extraction process. The dimensions of the chip were 45 mm × 15 mm × 1.25 mm. The extraction efficiency was 40.1%.

A method was successfully developed to integrate a DNA extraction matrix into glass microchannels. The sol-gel and silica bead matrices bind DNA/RNA through an electrostatic interaction. The silica beads provided a large surface area for selective binding of DNA, while the sol-gel serves as a silica-based glue to hold beads in place during device operation. The packed bed was 10 to 20 mm in length and the channel dimensions were 150 µm wide, 150 µm deep (Figure 5a). In Figure 5b, the device had several regions that were completely packed with sol-gel matrix and some regions that were less than optimal. The cross-section in Figure 5c is not rectangular due to the hydrofluoric acid etching process used in the fabrication (Micronit Microtechnologies, Enschede, The Netherlands).

Using the device in Figure 5a, a pre-wash step using MeOH was used to activate the silica, which primed the microchannels. Loading was performed using 25 µL of DNA in guanidine hydrochloride (250 fmol), which was flowed through the microchannels at 2 µL/min. The DNA bound to the silica matrix, allowing excess lysate and proteins to pass through as waste. The wash step was performed using 25 µL of 80% 2-propanol to remove excess guanidine hydrochloride and contaminants from the microchannels. DNA was eluted from the solid phase by flowing TE buffer through the microchannels. The load, wash, and eluent were collected in microcentrifuge tubes and analyzed using DNAQF DNA quantitation fluorescent assay kit. The black line is the amount of DNA at each stage of the extraction process where the red line is the sum of the DNA throughout the process. The extraction efficiency was 40.1% for the sol-gel packed microchannels.

### 3.3. DNA Extraction Using Paramagnetic Beads with Acoustic Actuation

Fabricated cartridges were evaluated for their ability to recover genomic *E. coli* DNA strain B (Sigma-Aldrich, St. Louis, MO, USA). To independently measure the lysing efficiency and the DNA extraction efficiency, a known amount of DNA was introduced into the microchannel shown in Appendix B, Figure A2a. Approximately 1 × 10^6^ beads were loaded into the channel to measure DNA extraction efficiency. This amount was verified using a flow cytometer to measure the bead concentration. The particles concentration prior to loading was compared with the collected eluent after loading the beads into the microchannel. It was determined that quantities greater than 1 × 10^6^ beads restricted fluid flow in the microchannel, and sufficient pressures could not be generated to push the fluid through the device without compromising the integrity of the cartridge.

**Figure 6:**
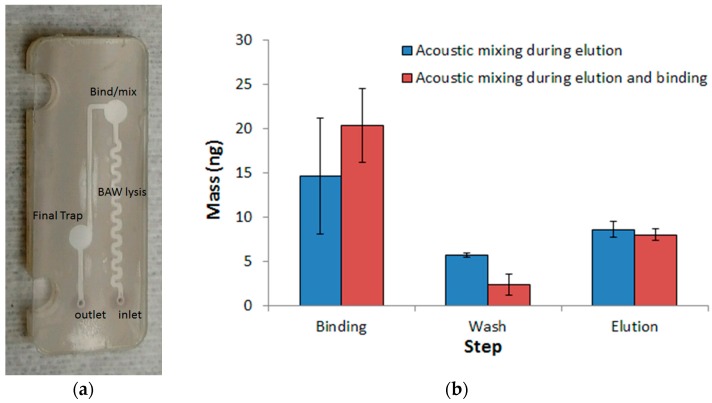
Nucleic acid binding magnetic bead-based cartridge and extraction. (**a**) Plastic laminate-based nucleic acid binding magnetic bead-based cartridge; (**b**) Mass of nucleic acid extracted using ChargeSwitch^®^ beads with microfluidic cartridges using a flow rate of 10 μL/min. The extraction efficiency was 39.4% for the acoustic mixing during elution and binding case and 58.9% for acoustic mixing during elution. *n* = 5, reported as mean ± std. dev mean.

Figure 6 compared the DNA extraction performance of the ChargeSwitch^®^ beads when 45 ng of DNA was loaded into the channel. The maximum binding capacity for 1 × 10^6^ beads is 50 ng of DNA. In the first trial, the bead trap/mixing region was located over an acoustic transducer. The 54 MHz acoustic transducer was driven at 135 mW, with a 25% duty cycle for mixing purposes only. Elsewhere, a duty cycle of 100% was used for the 54 MHz transducer array. Acoustic mixing was used during the elution step to facilitate mixing of the beads and removal of the DNA from the bead surface. In the second trial, acoustic actuation was applied to the trap/mixing region during binding and elution. The motivation was to enhance DNA-bead interaction rather than rely on diffusion. The acoustic transducer was driven at 25 mW for the binding step and 135 mW for the elution step. In Figure 6b, there was no significant improvement in DNA binding to the ChargeSwitch^®^ beads in the presence of acoustic mixing. The total amount of injected DNA was 45 ng, which yielded 20.4 ng bound to beads in the microchannels. After elution, the amount of recovered DNA was 8.0 ng for acoustic mixing during elution and binding, yielding an extraction efficiency of 8.0 ng/20.4 ng = 39.4% and for acoustic mixing during elution, the efficiency was 8.6 ng/14.6 ng = 58.9% (Figure 6). The amount of DNA eluted was similar for the two approaches. 

It was determined that a significant amount of DNA was lost to the adhesive regions in the microchannel (not shown), where only ~20% of the total DNA was recovered. Given that the cartridges were fabricated in plastic, the intrinsic surface charge binds DNA and causes sample loss to the wetted surfaces. Non-specific losses can be minimized by passivating the microchannel to reduce or block the surface charges, thereby decreasing DNA binding. However, passivating with proteins such as bovine serum albumin caused continual desorption of proteins from the wetted surfaces. This desorption problem may interfere with subsequent measurements. Another option is to use cyclic olefin polymer (COP), which has low nucleic acid and protein adsorption properties. This would require more advanced assembly techniques to bond the various COP layers together. These cartridges may be fabricated in glass using laminates like the one employed here, however, the bonding would require a thermal process (e.g., laser welding). Glass fabricated microchannels would also permit optical measurements and lower protein binding compared to some plastics.

### 3.4. DNA Extraction Using Nafion-Based Cartridge

**Figure 7:**
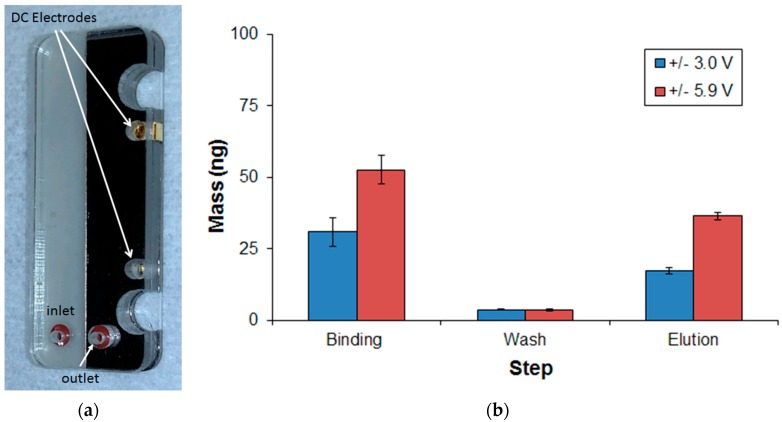
Nucleic acid Nafion-based cartridge and extraction. (**a**) Plastic laminate-based nucleic acid binding electrodes coated with Nafion; (**b**) Mass of nucleic acid extracted using a flow rate of 10 µL/min. The extraction efficiency was 69.2% at a bias of ±5.9 V.

The third method studied for nucleic acid extraction used an electric field to trap nucleic acid to a Nafion passivated gold electrode. This approach was based on preliminary results that showed passivation with Nafion maintained integrity of the nucleic acid during binding while preventing electrode degradation. It is likely that Nafion films (i.e., perfluorinated resin) preserve biomolecule integrity by preventing nucleic acid from reaching the highly reactive electrode surface. The pores in the Nafion film were sufficiently small to prevent DNA migration while allowing ion mobility of cations to the electrode surface. The thickness of the Nafion film was 12 µm. Using the assembled lysing/Nafion cartridges shown in Figure 7, the amount of DNA was measured during binding, wash, and elution steps for two applied voltages. The DNA extraction efficiency was computed as the amount of DNA bound to the Nafion film during the binding step and not the injected amount of DNA. The total amount of injected DNA was 90 ng, which yielded 54.8 ng bound to the electrodes at a voltage of 5.9 V. The injected amount of 90 ng was discovered to exceed the binding capacity of the Nafion passivated Au electrodes. To calculate the DNA extraction efficiency, the amount of DNA bound to the electrodes was defined as 100%. Thus, 37.9 ng/54.8 ng = 69.2% extraction efficiency at ±5.9 V.

Maintaining the DC bias voltage during the wash step was crucial to prevent excessive DNA loss. An extraction efficiency of 56.3% was measured for an applied bias voltage of ±3.0 V during the binding and wash steps. When ±5.9 V was applied, the amount of DNA binding increased significantly to 69.2%. However, at voltages exceeding 6 V, the DNA binding did not increase any further. Other groups have reported DNA extraction efficiencies as high as 70–80% in a miniature format using silica resins [6,7] and greater than 70% for Nafion-coated electrodes [35]. In terms of state-of-the-art (SoA), extraction efficiencies observed in this study commensurate with previous efforts, with the exception of the sol-gel packed bead microchannels. The disparity is probably due to the low-packing density of the sol-gel packed beads in the microchannel compared to a packed-bead matrix.

## 4. Conclusions

In this work, a miniature BAW transducer array was presented that rapidly releases the internal contents of cells, without involving chemicals or other interferences. The BAW transducer array was integrated with disposable plastic cartridges and coupled to a sol-gel packed microchannel to capture and purify nucleic acid. Three types of nucleic acid extraction approaches were studied: sol-gel silica bead matrices, paramagnetic nucleic acid binding beads, and an electric field extraction using Nafion films on gold electrodes. In terms of applied energy (power × treatment time), the BAW array used 225× less and 23,000× less than the finger and cup methods, respectively. This was due to the higher frequency of the miniature BAW array since 600× more sonication cycles were used. The ability to lyse cells using plastic-based microchannels was also demonstrated, which substantially simplifies fabrication complexity and cost compared to microchannels fabricated from glass. Though plastic laminates provided a simple way to test complex geometries, the adhesives used in the bonding process contributed to DNA loss. This can be reduced substantially by using low-binding plastics such as cyclic olefin polymers (COP), which have fully saturated backbones, and hence are very stable. The Nafion method proved to have the highest extraction efficiency amongst the three methods. All the methods would benefit from using low-binding materials to reduce non-specific loss to the microchannels. It is anticipated that this technology will have wide application in existing biosensors and offer excellent scalability for multi-analyte detection applications. 

## Figures and Tables

**Figure A1:**
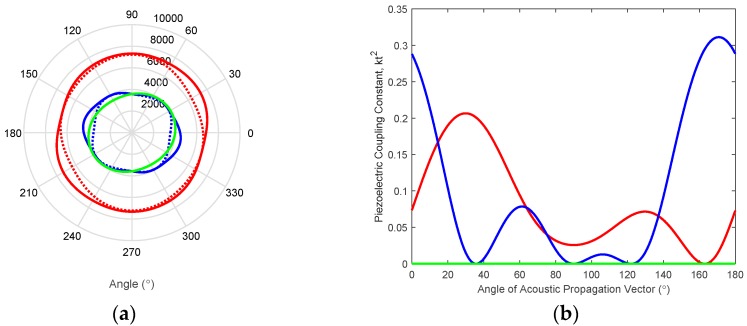
The acoustic velocity and electromechanical coupling $k_{t}^{2}$ for 36° Y-cut (90°, 90°, 36°) lithium niobate. (**a**) Acoustic velocities for the piezoelectricity case (solid line) and non-piezoelectric case (dotted line), (red) longitudinal, (green) fast shear mode, and (blue) slow shear mode and; (**b**) the thickness mode electromechanical coupling.

## Appendix A

The acoustic velocity and electromechanical coupling of 36° Y-cut lithium niobate was calculated by solving the Christoffel equation for anisotropic wave propagation in solids [36]:

(A1) $$\rho v^{2}u_{j}=\left(l_{ik}\left[c_{KL}^{E}+\frac{\left(e_{Kj}l_{j}\right)\left(l_{i}e_{iL}\right)}{l_{i}\varepsilon_{ij}^{S}l_{j}}\right]l_{Lj}\right)u_{j}$$

where $c_{KL}^{E}$ is the symmetric 6 × 6 elastic stiffness tensor (N/m^2^) of rank 4, hence with 81 elements, but it reduces to 21 independent stiffness variables, ***e_Kj_*** (6 × 3) and ***e_iL_*** (3 × 6) are the piezoelectric tensors (C/m), $\varepsilon_{ij}^{S}$ is the permittivity tensor (3 × 3) (F/m) at constant electric strain, ρ is the density, *v* are the acoustic phase velocities, ***l*** is the propagation matrix (***l_iK_****:* 3 × 6, ***l_Lj_***: 6 × 3, ***l_i_***: 1 × 3, and ***l_j_***: 3 × 1), and ***u*** is the polarization vector. The solution to the Christoffel equation yields three eigenvalues or phase velocities of the three wave modes that can exist in an anisotropic solid. Each eigenvalue has a corresponding eigenvector describing the displacement caused by each mode. Though (A1) is easily solved using eigenvalue methods, the solutions must be sorted to distinguish longitudinal modes from the fast and slow shear modes. The normalized polarization vector (***p***) is the wave modes are:

(A2) $$p_{i}=\frac{u_{i}}{\left|u_{j}\right|}$$

When the polarization vector is parallel to the normal direction of the wavefront, the wave is a pure longitudinal mode (*L*). The wave is pure shear (*S*) when the polarization vector is perpendicular to the wavefront normal direction. If the polarization vector is neither of these conditions, the wave propagates in a quasi-mode (QM). The electromechanical coupling ($k_{t}^{2}$) is defined as [36]:

(A3) $$k_{t}^{2}=\frac{e_{ij}^{2}}{\varepsilon_{ij}^{S}c_{ij}^{D}}=\frac{K^{2}}{1+K^{2}}$$

where the piezoelectric coupling constant is [36]:

(A4) $$K^{2}=\frac{e_{ij}^{2}}{\varepsilon_{ij}^{S}c_{ij}^{E}}=\frac{e_{ij}^{2}}{\varepsilon_{ij}^{S}(c_{ij}^{D}-\frac{e_{ij}^{2}}{\varepsilon_{ij}^{S}})}=\frac{v_{p}^{2}}{v_{np}^{2}}-1$$

where *v_p_* is the acoustic phase velocity with piezoelectricity and *v_np_* is the acoustic velocity in the absence of piezoelectricity. The presence of piezoelectricity stiffens *c^E^* by the term *e*^2^/ε*^S^*. The expression on the r.h.s of (A4) is an approximation. Solving (A1) for the acoustic velocities and using (A2) for the polarizations, the electromechanical coupling was computed using (A3) and (A4). The fully automated program solved and sorted the acoustic modes based on their polarizations. The acoustic velocities and thickness mode electromechanical coupling are shown in Figure A1.

## Appendix B

### Appendix B.1. Fabrication of Acoustic Lysis and Paramagnetic Bead-Based Nucleic Acid Extraction Cartridges

The lysis and nucleic acid extraction microsystem was fabricated from layers of fused silica, Mylar^®^ and polymethylmethacrylate (PMMA) (Fralock, Valencia, CA, USA) using a VersaLaser VLS3.50 system (Universal Laser Systems, Scottsdale, AZ, USA). The extraction approach is shown in Figure 2b, where the laminate cartridge lyses cellular samples and immediately captures nucleic acid to the surface of paramagnetic beads. The magnetic core of the beads trapped them within the microchannel as the buffer was exchanged to remove non-nucleic material. ChargeSwitch^®^ gDNA Mini Bacteria Kit manufactured by Invitrogen^TM^ (Carlsbad, CA, USA) was used to bind DNA reversibly. The beads bind DNA in response to a change in the solution pH. Following cell lysis, DNA was bound to the surface of the 1 μm ChargeSwitch^®^ magnetic beads by decreasing the solution pH below 6.5, which protonates the bead surface for binding with the negatively charged phosphate backbone of the DNA. The ChargeSwitch^®^ kit contained all the reagents to bind, rinse, and elute DNA from the ChargeSwitch^®^ magnetic beads. Bead loading efficiency was determined by injecting a known bead suspension into the microfluidic channel with the magnets engaged and performing a particle count on the eluent using an Accuri C6 Flow Cytometer (Accuri Cytometers, Ann Arbor, MI, USA). For these experiments, the buffers will be referred to as “binding buffer” (pH < 6.5), “wash buffer” (pH = 7.0), and “elution buffer” (pH = 8.5). For each experiment, 100 μL of genomic *E. coli* genomic DNA solution was combined with 300 μL of binding buffer and injected into the microfluidic cartridge, followed by 500 μL of wash buffer to remove nonspecifically bound impurities. In the last step, 200 μL of elution buffer was injected to release bound DNA. The eluent from each step was collected and analyzed for DNA content using a Quant-iT™ PicoGreen assay purchased from Invitrogen (Carlsbad, CA, USA). PicoGreen is a dye that fluoresces upon binding with double-stranded DNA. The assay was performed in a 96-well plate format using a SpectraMax M2 plate reader (Molecular Devices, Sunnyvale, CA, USA) and the data fitted to a standard curve generated from known concentrations of DNA.

In Figure A2a, a piezoelectric transducer was positioned under the Trap/Mixing reservoir for active mixing of the beads with the reagent solution. This process enabled removal of nucleic acid from surface of the beads using acoustic waves during the elution step. In addition, 3.2 mm diameter magnets served to trap beads within the microchannels (KJ Magnetics, Jameson, PA, USA). Unbound contaminants were removed in two wash steps, and the DNA was eluted from the beads by raising the pH to 8.5, which deprotonates the bead surface and releases the bound DNA. The paramagnetic core of the beads permitted immobilization of the beads within an externally applied magnetic field for reagent exchange with minimal sample loss. The microchannels were 100 µm in height and 500 µm in width. Cells were lysed in the region defined in Layer #2 and then the nucleic acid-bead complex was trapped by a magnetic field (Figure A2a). Acoustic actuation was used to improve the removal of nucleic acid from the paramagnetic beads in the presence of the elution buffer. The magnetic trap was 0.125” in diameter. In Figure A2a, the O-ring layer (i.e., layer #6) held a 001 silicone O-ring (I.D. 1/32”, O.D. 3/32”) (McMaster-Carr, Los Angeles, CA, USA). A final magnetic trap was used to prevent bead loss from the microchannel cartridge. The stack was assembled using alignment holes in each layer and then compressed in a hydraulic press for 2 min at 2700 kg.

### Appendix B.2. Fabrication of Nafion-Based DNA Extraction Cartridges

The Nafion-based nucleic acid extraction cartridge is shown in Figure A2a. Using an electric field-based nucleic acid extraction concept presented by Lee et al. [35], acoustic lysis and nucleic acid extraction were integrated into a single disposable cartridge. The top layer was fabricated using 62 mil thick PMMA to provide strain relief for the tubing connections. The tubing (fluorinated ethylene propylene (FEP) 1 mm O.D., 0.5 mm I.D., Upchurch Scientific, Oak Harbor, WA, USA). This cartridge was assembled using two sub-assemblies, (1) layers 2–8 were bonded together to create the lysis and nucleic acid binding region and (2) the fused silica layer was bonded separately at lower pressure ~100 psi. During assembly, layer 6 defined a unique wrap-around electrode that bonded to layer 8 and was held in place by layer 9. Layers 2–8 were compressed for 2 min at 2700 kg. The microchannels were 100 µm in height and 500 µm in width. The electrodes were fabricated by evaporating 200 Å of Cr and 5000 Å of Au onto 100 µm thick Mylar^®^ discs. The discs were used to create layers 4 and 6 in Figure A2b. This permitted single-sided electrical connectivity to the Nafion-coated electrodes. The available surface area for nucleic acid binding was 141 mm^2^. For each experiment, 50 μL of *E. coli* genomic DNA in 10 mM TE buffer (10 mM Tris-Cl pH 7.4, 1 mM EDTA pH 8.0) was injected into the microfluidic channel at a 50 μL/min. flow rate. Upon solution entering the channel, a positive voltage was applied to the electrode using an Agilent E3630A DC power supply to bind nucleic acid. A volume of 50 μL of 10 mM TE buffer was loaded into the channel at 50 μL/min flow rate, and a positive voltage was applied to the electrode to bind nucleic acid until the entire volume of buffer had exited the channel. The voltage was held constant during the wash step. For the elution step, 50 μL of 10 mM TE buffer was loaded into the channel at 50 μL/min and a negative voltage was applied to release bound DNA from the electrode. The eluent from each step was collected and analyzed for DNA content using the Quant-iT™ PicoGreen assay. The voltages for binding and elution of DNA were chosen based on electric field calculations and ranged from ±2.5 V to ±7.9 V.
